# The effects of hypoxia on muscle deoxygenation and recruitment in the flexor digitorum superficialis during submaximal intermittent handgrip exercise

**DOI:** 10.1186/s13102-020-00163-2

**Published:** 2020-05-15

**Authors:** Hayley J. Nell, Laura M. Castelli, Dino Bertani, Aaron A. Jipson, Sean F. Meagher, Luana T. Melo, Karl Zabjek, W. Darlene Reid

**Affiliations:** 1 Department of Physical Therapy, University of Toronto, 160-500 University Avenue, Toronto, ON M5G 1V7 Canada; 2 KITE, Toronto Rehab-University Health Network, 550 University Ave, Toronto, ON M5G 2A2 Canada; 3 Interdepartmental Division of Critical Care Medicine, University of Toronto, Li Ka Shing Knowledge Institute, 209 Victoria Street, 4th Floor, Room 411, Toronto, ON M5B 1T8 Canada

**Keywords:** Near-infrared spectroscopy, Hypoxia, Exercise, Skeletal muscle

## Abstract

**Background:**

Decreased oxygenation of muscle may be accentuated during exercise at high altitude. Monitoring the oxygen saturation of muscle (SmO_2_) during hand grip exercise using near infrared spectroscopy during acute exposure to hypoxia could provide a model for a test of muscle performance without the competing cardiovascular stresses that occur during a cycle ergometer or treadmill test. The purpose of this study was to examine and compare acute exposure to normobaric hypoxia versus normoxia on deoxygenation and recruitment of the flexor digitorum superficialis (FDS) during submaximal intermittent handgrip exercise (HGE) in healthy adults.

**Methods:**

Twenty subjects (11 M/9 F) performed HGE at 50% of maximum voluntary contraction, with a duty cycle of 2 s:1 s until task failure on two occasions one week apart, randomly assigned to normobaric hypoxia (FiO_2_ = 12%) or normoxia (FiO_2_ = 21%). Near-infrared spectroscopy monitored SmO_2_, oxygenated (O_2_Hb), deoxygenated (HHb), and total hemoglobin (tHb) over the FDS. Surface electromyography derived root mean square and mean power frequency of the FDS.

**Results:**

Hypoxic compared to normoxic HGE induced a lower FDS SmO_2_ (63.8 ± 2.2 vs. 69.0 ± 1.5, *p* = 0.001) and both protocols decreased FDS SmO_2_ from baseline to task failure. FDS mean power frequency was lower during hypoxic compared to normoxic HGE (64.0 ± 1.4 vs. 68.2 ± 2.0 Hz, *p* = 0.04) and both decreased mean power frequency from the first contractions to task failure (*p* = 0.000). Under both hypoxia and normoxia, HHb, tHb and root mean square increased from baseline to task failure whereas O_2_Hb decreased and then increased during HGE. Arterial oxygen saturation via pulse oximetry (SpO_2_) was lower during hypoxia compared to normoxia conditions (*p* = 0.000) and heart rate and diastolic blood pressure only demonstrated small increases. Task durations and the tension-time index of HGE did not differ between normoxic and hypoxic trials.

**Conclusion:**

Hypoxic compared to normoxic HGE decreased SmO_2_ and induced lower mean power frequency in the FDS, during repetitive hand grip exercise however did not result in differences in task durations or tension-time indices. The fiber type composition of FDS, and high duty cycle and intensity may have contributed greater dependence on anaerobiosis.

## Introduction

Optimal oxygen delivery and utilization by skeletal muscle is essential to maximize muscle performance and exercise capacity. It is well established that poor delivery of oxygen to muscles greatly compromises performance and exercise capacity [1, 2]. Previous studies have demonstrated that acute exposure to hypoxia impairs muscle endurance and performance in large muscle groups [3, 4]. Similarly, the effects of hypoxia on smaller muscle groups has also been associated with reduced muscular endurance and performance, albeit to a lesser degree [5]. High-intensity intermittent static contractions of adductor pollicis muscle resulted in reduced endurance times during hypobaric hypoxia compared with normoxia [5–7]. A review by Perrey & Rupp outlined that acute hypoxic exposure when compared to normoxic conditions leads to a decline in muscular endurance time when protocols employed submaximal intermittent isometric contractions [8]. On the other hand, acute hypoxia exposure on maximal voluntary force generating capacity of small muscle groups appears to have minimal to no reduction in force production and the rate of decline of force compared to normoxic conditions [6, 9].

Near-infrared spectroscopy (NIRS) can estimate muscle oxygenation during exercise via continuous-wave emissions of near-infrared at approximately 760 nm and 850 nm, which is absorbed by deoxygenated (HHb) and oxygenated hemoglobin (O_2_Hb), respectively. The change in concentration of these chromophores can be estimated from the modified Beer-Lambert law, which compensates for light scattering during emission through tissues [10]. Using spatially resolved NIRS, the saturation of muscle oxygenation (SmO_2_) can be quantified [11]. NIRS has been shown to be reliable [12], and valid to measure muscle oxygenation at rest [13] and during exercise [14, 15]. Its wireless, non-invasive application provides an unobtrusive tool for measuring muscle oxygenation to further explore the range of deoxygenation to improve muscle function while minimizing adverse effects.

Muscle oxygenation and deoxygenation profiles in small muscle groups such as the forearm and finger flexors is of particular relevance to the climbing population, where finger and grip strength is of upmost importance. Previous studies in climbers examining muscle oxygenation in small muscle groups during continuous and intermittent isometric testing protocols have shown a greater level of muscle deoxygenation and faster rates of reoxygenation in elite climbers compared to controls [16–18]. When examining muscle oxygenation profiles in small muscle groups during acute exposure to hypoxia, variable responses have been noted [19–21]. A study by Hicks et al., demonstrated that muscle oxygenation of forearm muscles during a 30% isometric MVC did not differ between hypoxia (FiO_2_ = 14%) and normoxia [19]. In contrast, Hansen et al. found that hypoxia (FiO_2_ = 10%) decreased O_2_Hb in the flexor digitorum profundus during intermittent handgrip exercise at 5% of MVC, with a 50% duty cycle for a total of 5 min [22]. The variable effect of hypoxia on muscle oxygenation profiles may in part be due to the type of contraction used (intermittent or sustained isometric). In addition, other factors such as the duty cycle, intensity, and muscle could influence outcomes.

Few studies to date have investigated the effects of acute exposure to hypoxia on forearm muscle oxygenation and muscle performance during higher duty cycles of submaximal intermittent isometric exercise to task failure. Moreover, concurrent muscle oxygenation and activation are not often reported in the forearm muscles during exercise under hypoxic compared to normoxic conditions. Focusing on upper body musculature, in particular forearm flexors, is of relevance considering their significant contribution to grip strength [23], which is required for many daily tasks [24] and is also relevant to athletes involved in sports with repetitive gripping, such as for bouldering, rock climbing [25] and martial arts [26]. Additionally, investigating a small muscle group allows for examination of the effects of acute exposure to hypoxia without the competing cardiovascular stresses that occur during a cycle ergometer or treadmill test. Utilizing sEMG and NIRS simultaneously allows non-invasive evaluation of muscle recruitment complemented by muscle oxygenation, which can provide important insight into both the activation and metabolic state of the working muscle that might limit exercise.

Therefore, the purpose of this study was to characterize the effects of normobaric hypoxia during submaximal intermittent handgrip exercise (HGE) (50% MVC, work to rest ratio of 2 s:1 s) to task failure on muscle deoxygenation and motor unit recruitment of flexor digitorum superficialis (FDS) in healthy subjects. We hypothesized that FDS SmO_2_ and O_2_Hb would decrease more so during hypoxic compared to normoxic HGE. Secondly, we postulated that there would be increased FDS motor unit recruitment and decreased firing rate (via sEMG) under hypoxic compared to normoxic HGE.

## Methods

### Participants and screening

Twenty-four participants were screened from the general community at the University of Toronto. Inclusion criteria were: healthy men and women, aged 18–40 years, non-smokers and sufficient fluency in English to understand instructions and provide informed consent. Participants were excluded if they: had adipose tissue thickness > 10 mm over target muscle for NIRS monitoring, were elite athletes (participated at university, provincial, or national level competition in the past year), were anemic, had unresolved upper extremity injury or condition on their dominant side, or had forced expiratory volume in 1 s (FEV_1_) and/or forced vital capacity (FVC) percent predicted values < 80%. Of the 24 participants screened, two did not meet the inclusion criteria; one was identified as a university athlete and the other had an FEV_1_ lower than 80% percent predicted. All experimental procedures and protocols were approved by the University of Toronto clinical research ethics board (Protocol ID 32301).

### Experimental design

**Fig. 1:**
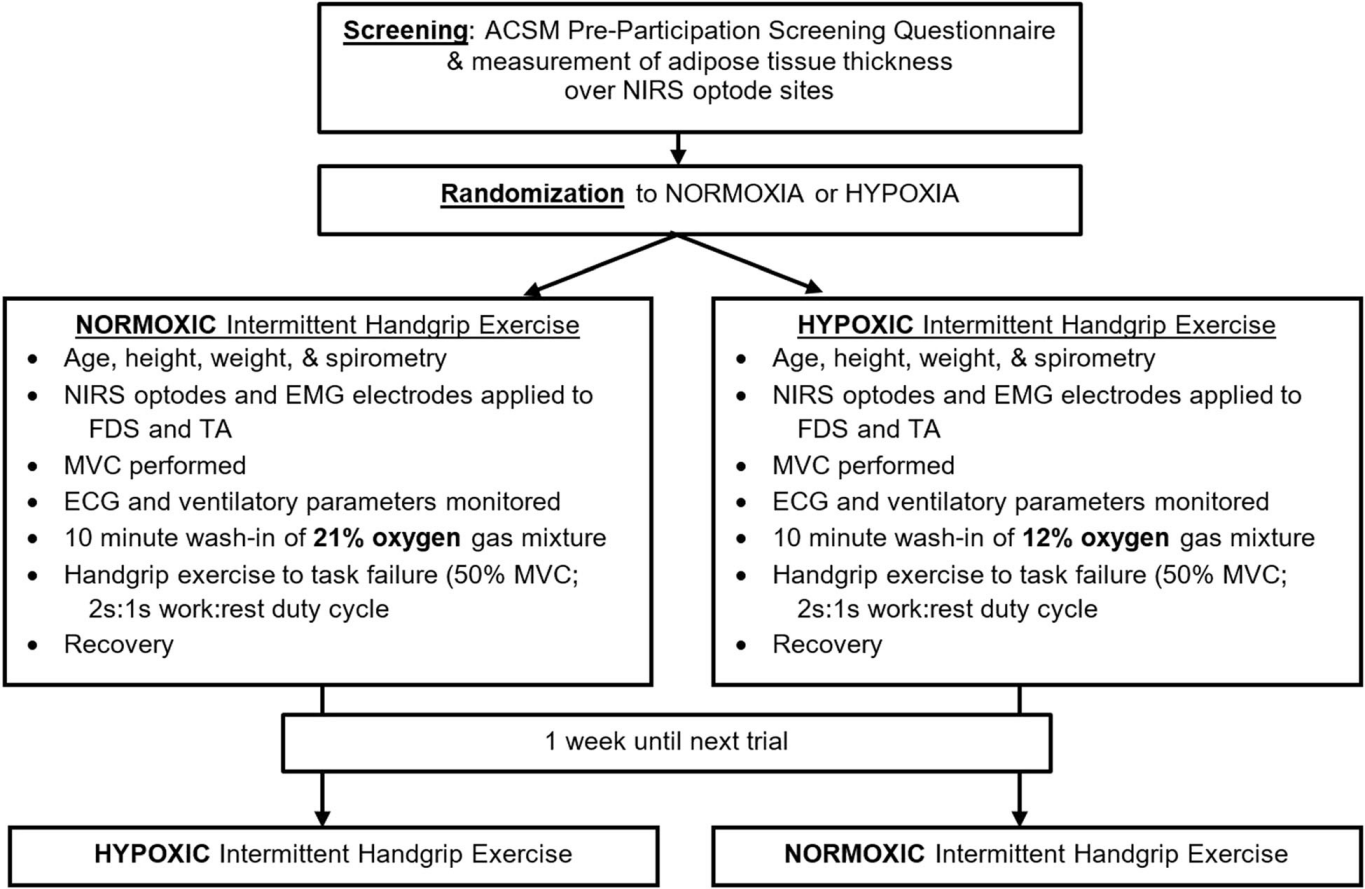
Experimental protocol. *ECG* electrocardiogram, *EMG* electromyography, *FDS* flexor digitorum superficialis, *MVC* maximal voluntary contraction. *NIRS* near-infrared spectroscopy, *TA* tibialis anterior

This study was a randomized, double-blind, repeated measures design. Figure 1 depicts an overview of the experimental protocol. Verbal and written explanation of the study was provided and written informed consent was obtained from participants. All participants were screened for physical readiness to engage in exercise using the American Heart Association and American College of Sports Medicine Pre-Participation Screening Questionnaire [27]. Participants refrained from exercising on trial days, and abstained from caffeine and alcohol consumption 12 and 24 h prior to the trial. FEV_1_ and FVC were measured with a spirometer (COPD-64000, Vitalograph, Ennis, Ireland) according to ATS/ERS standards [28]. Adipose thickness over FDS and tibialis anterior (TA) were determined using ultrasound (GE LOGIQ e portable USI), to rule out participants with adipose tissue thickness > 10 mm over target muscles [29]. As the fourth digit contributes to 25 to 28% of total power grip force [23, 30], the FDS muscle belly of the fourth digit on the dominant arm was landmarked by resisted fourth digit flexion [30]. The control muscle, TA, was landmarked by determining one-third the distance between the fibular head and medial malleolus and confirmed by palpation with active dorsiflexion in sitting.

Participants performed two HGE trials one week apart, while they were blinded to normoxic and normobaric hypoxic conditions that were randomly ordered. Investigators acquiring and analyzing NIRS and EMG outcomes were also blinded to the gas condition. Participants inspired either a mixture of medical grade gas corrected by nitrogen to a fraction of inspired oxygen (FiO_2_) of 12% (PiO2 ~ 11.5 kPa) for the normobaric hypoxic condition, or 21% (PiO2 ~ 20.0 kPa) for the normoxic condition. Participants wore nose plugs and breathed through a flanged mouthpiece connected to a 2-way Hans-Rudolph valve with the expiratory port to room air. The inspiratory port was attached via large bore tubing to a 150 L non-rebreathing bag filled with the normobaric hypoxic or normoxic gas mixture. Participants inspired the gas for a ten-minute wash-in period prior to the HGE protocol, to allow equilibrium of the gas concentrations between the lungs and inspired gas mixture. The partial pressure of end-tidal CO_2_ (P_ET_CO_2_) (ML206, ADInstruments), breathing frequency, and tidal volume were monitored throughout the trial duration (PowerLab, ADInstruments; Colorado Springs, CO). Heart rate was monitored using 3-lead electrocardiogram, while SpO_2_ was measured using an oximeter (8000AA, Nonin, Plymouth, MN). Blood pressure was measured before and after each trial (Blood Pressure Monitor 106–964, AMG Physiologic, West Chazy, NY). Rating of perceived exertion for dyspnea and forearm fatigue was obtained using a 10-point Borg scale before wash-in and immediately after task failure for both trials.

Prior to commencement of the first trial, MVC of handgrip force for the participants dominant hand was determined using the grip force transducer (MLT004/ST, ADInstruments, Colorado Springs, CO). Participants completed three MVCs, with a two minute separation between contractions. The highest force produced was recorded as the MVC, and 50% MVC ± 5% was calculated. Unit markers were added on the computer monitor through the LabChart software to indicate a target range of 45 to 55% of MVC for participants to use for visual feedback. Prior to wash-in and before each trial, five practice contractions were completed. Temporal synchronization between the PowerLab data acquisition unit and Oxysoft software (Oxysoft 3.0.95, Artinis Medical Systems, Elst, Netherlands) for NIRS data was achieved using a bluetooth synchronization device (Portasync, Artinis Medical Systems). Participants were required to grip the transducer with a force equal to 50% MVC ± 5% and a work to rest ratio of 2:1, equating to a two second grip contraction period and a one second rest period. A pre-recorded audio message guided the participant through the protocol; no additional verbal encouragement was provided throughout the trial. Participants performed the protocol until task failure, as indicated by the inability to reach target % MVC for three consecutive contractions [31]. Tension-time index in Newton-minutes, a measure of force-time product, was calculated as the integral of all contractions using the LabChart software.

### Near-infrared spectroscopy

Levels of SmO_2_ (%) and changes of O_2_Hb, HHb, tHb (μM) of FDS and TA were monitored using NIRS during HGE from baseline (last minute of wash-in period) until task failure while breathing hypoxic or normoxic gas. NIRS optodes of the PortaLite mini and PortaLite (Artinis Medical Systems, Elst BV, Netherlands) were placed over the dominant FDS and TA, respectively. The PortaLite mini and PortaLite devices contain three light sources and one receiver with interoptode distances of 16, 21, and 26 mm and 30, 35, and 40 mm, respectively. The differential pathlength factors (DPF) was set at 4 and data was acquired at 10 Hz. The change in NIRS variables was determined as the difference between the baseline values during the last 5 s compared to the mean of the last three contractions immediately before task failure. O_2_Hb, HHb, and tHb were derived from the optode pair (emitter-receiver) that showed the greatest changes.

### Electromyography

Surface electromyography was used to determine the electrophysiological response of FDS to hypoxic and normoxic conditions during a HGE. The skin over the FDS was shaved and swabbed with alcohol prior to electrode placement. Gel electrodes (Kendall Medi-Trac Mini 130, King Medical, London, ON, Canada) were placed distal to the NIRS optodes and in line with the muscle fibers of FDS. A ground electrode was placed over the C7 spinous process. Appropriate electrode placement was confirmed by gripping the grip force transducer (MLT004/ST, ADInstruments, Colorado Springs, CO), and obtaining an adequate signal from the PowerLab data acquisition unit (ADInstruments, Colorado Springs, CO) as determined by analyzing the signal using LabChart 8 data analysis software (ADInstruments, Colorado Springs, CO). A band-pass filter was applied to the raw sEMG signal with a high-pass of 400 Hz and a low-pass of 20 Hz and data was acquired at 2000 Hz. The root mean square measured in mV and mean power frequency measured in Hz was calculated using the LabChart software to isolate 1 s segments of sEMG signal from one of the first three contractions and one of the final three contractions during the HGE.

### Data analysis

Statistical analyses were performed using Statistical Package for Social Sciences (version 24.0, SPSS Inc., Chicago, IL). Paired sample t-tests were used to compare tension-time index (a measure of total workload, N∙min) and task duration between normoxic and hypoxic HGE. To analyze the changes in NIRS measurements, the total task duration of the handgrip protocol was divided into five quintiles (0–20, 20–40, 40–60, 60–80, 80–100%). Changes in O_2_Hb, HHb and tHb from baseline to each quintile of the handgrip task duration were determined. NIRS and sEMG values were tested for normality using the Shapiro-Wilk test. Two-way repeated measures ANOVAs were used to detect differences in the effect of gas mixture and trial duration (at each quintile from baseline to task failure) on SmO_2_, ΔO_2_Hb, ΔHHb and ΔtHb in the FDS and TA, and to detect changes in root mean square, mean power frequency and cardiorespiratory parameters (blood pressure, minute ventilation, P_ET_CO_2_, SpO_2_) from baseline to task failure. Data was tested for sphericity with Mauchly’s test and if necessary was corrected using the Greenhouse-Geisser method. If ANOVA showed a significant effect, the Bonferroni post-hoc test was performed to determine significant pairwise comparisons. The relationship between SmO_2_ and SpO_2_ was investigated using a Pearson correlation. A *P* value of < 0.05 was considered statistically significant. Data are reported as means ± standard deviation (SD) unless otherwise stated.

## Results

Twenty of 22 healthy participants completed the protocol; one was unable to reliably perform repetitive HGE to a 50% MVC target, and one participant’s NIRS values contained a high degree of artifact. Thus, 11 men and 9 women, with a mean age of 25 ± 3 years, and normal spirometry values (Table 1) performed the HGE protocol to completion under both hypoxic and normoxic conditions.

**Table 1:** Anthropometric and spirometry data of participants

Measure	Mean ± SD
Age	25 ± 3
Height (m)	172 ± 10
Mass (kg)	70.1 ± 12.0
BMI (kg/m^2^)	23.5 ± 2.7
FVC (%predicted)	88.0 ± 7.2
FEV_1_ (%predicted)	94.5 ± 6.6
FEV_1_/FVC	92.7 ± 8.3
Adipose tissue thickness over TA (mm)	2.0 ± 1.0
Adipose tissue thickness over FDS (mm)	3.1 ± 1.1
MVC (N)	610.3 ± 166.5

Data are expressed as mean ± SD (*n* = 20). *BMI* body mass index, *FVC* forced vital capacity

*FEV*_*1*_ forced expiratory volume in 1 s, *TA* tibialis anterior, *FDS* flexor digitorum

Superficialis, *MVC* maximal voluntary contraction

**Fig. 2:**
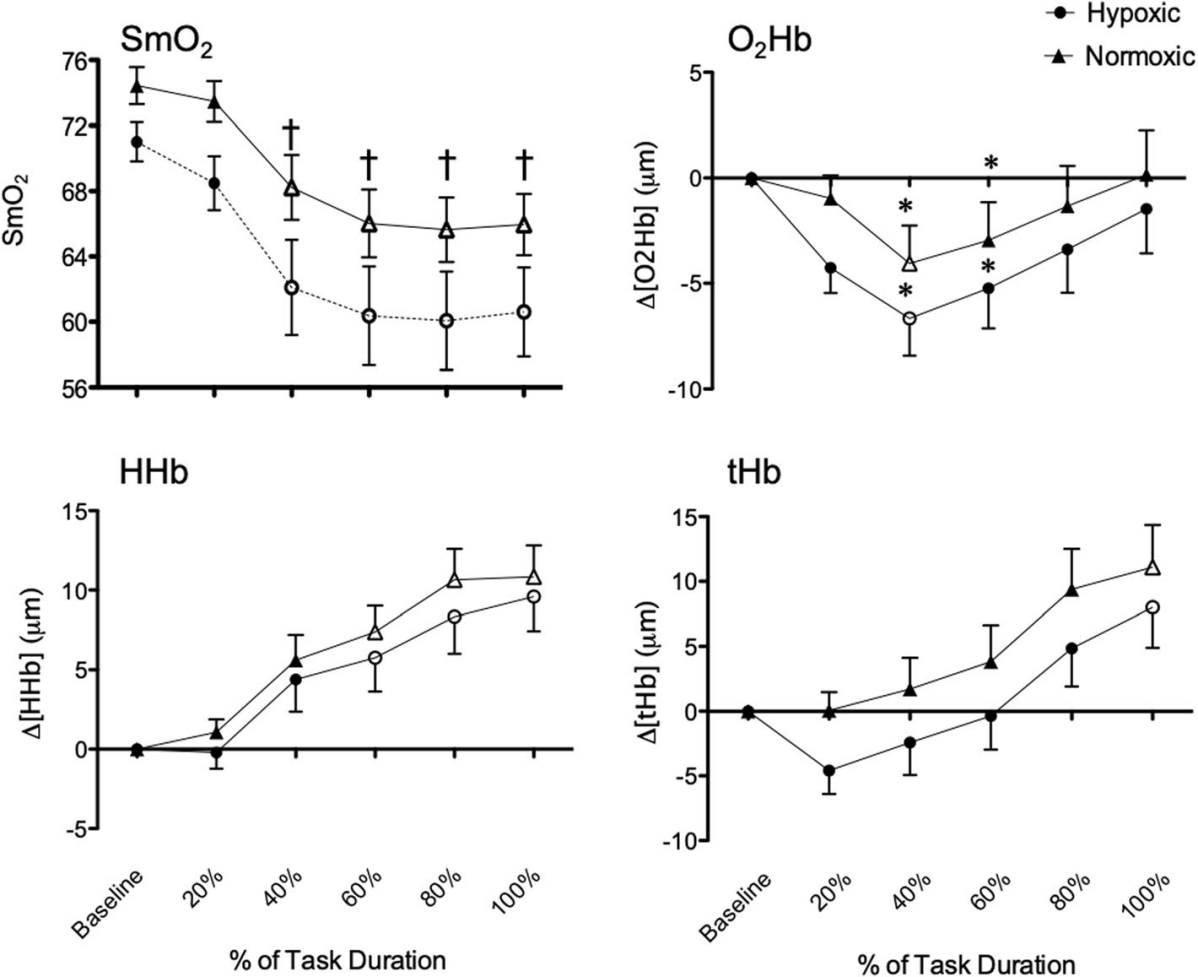
Muscle saturation of oxygen (SmO_2_) and changes in oxy- (ΔO_2_Hb), deoxy- (ΔHHb) and total hemoglobin (ΔtHb) during each quintile of hand grip exercise (HGE) from baseline until task failure (100%) in the flexor digitorum superficialis under hypoxic and normoxic conditions. Values are presented as mean ± SEM. Open markers indicate significant differences from baseline, asterisks denote significant differences from task failure, † indicates significant difference between hypoxic and normoxic conditions (*p <* 0.05)

**Fig. 3:**
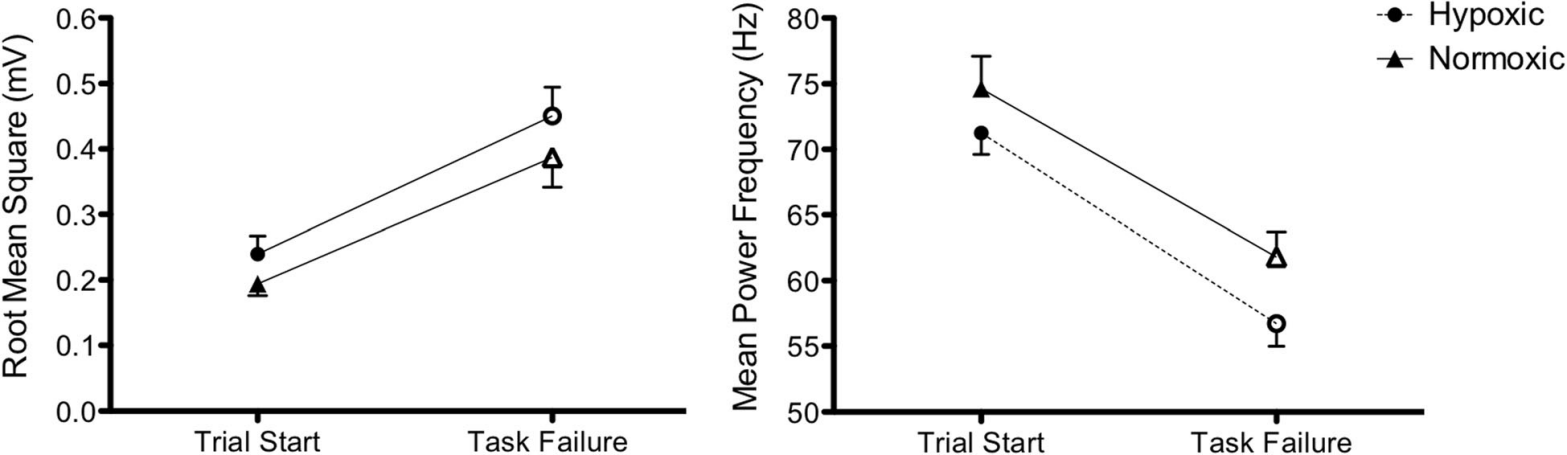
Root mean square and mean power frequency at the start of the trial and task failure during hand grip exercise (HGE) in the flexor digitorum superficialis. Trial start and task failure represent 1 s segments of sEMG signal from one of the first three contractions and one of the final three contractions during the HGE, respectively. Values are presented as mean ± SEM. Open markers indicate significant differences from trial start, dashed line indicates significant differences between gases (*p* < 0.05)

Hypoxic HGE resulted in a lower FDS SmO_2_ compared to normoxic HGE overall (63.8 ± 2.2 vs. 69.0 ± 1.5, *p* = 0.001) and at each quintile of duration, except 20% (Fig. 2). FDS O_2_Hb decreased from baseline to 40th percentile of task duration (*p* = 0.008) followed by an increase in FDS O_2_Hb from 40th percentile of task duration to task failure (*p* = 0.001) during hypoxic and normoxic HGE. Compared to baseline, FDS HHb increased at 60th percentiles of duration to task failure (*p* < 0.011). FDS tHb increased over time for both conditions with significant differences at task failure compared to baseline (*p* = 0.000). SmO_2_, O_2_Hb and tHb in the control muscle, TA, did not differ between gases or throughout the duration of the HGE. The only differences found in the control muscle was a small overall decrease in TA HHb during hypoxia compared to normoxia (*p* = 0.049). The root mean square values for FDS increased from the first contractions to task failure under both hypoxia and normoxia (*p* = 0.000) (Fig. 3). FDS mean power frequency decreased (*p* = 0.000) from the first contractions to task failure, and was lower during hypoxia compared to normoxia (63.99 ± 1.41 vs. 68.21 ± 1.99, *p* = 0.04) (Fig. 3).

**Fig. 4:**
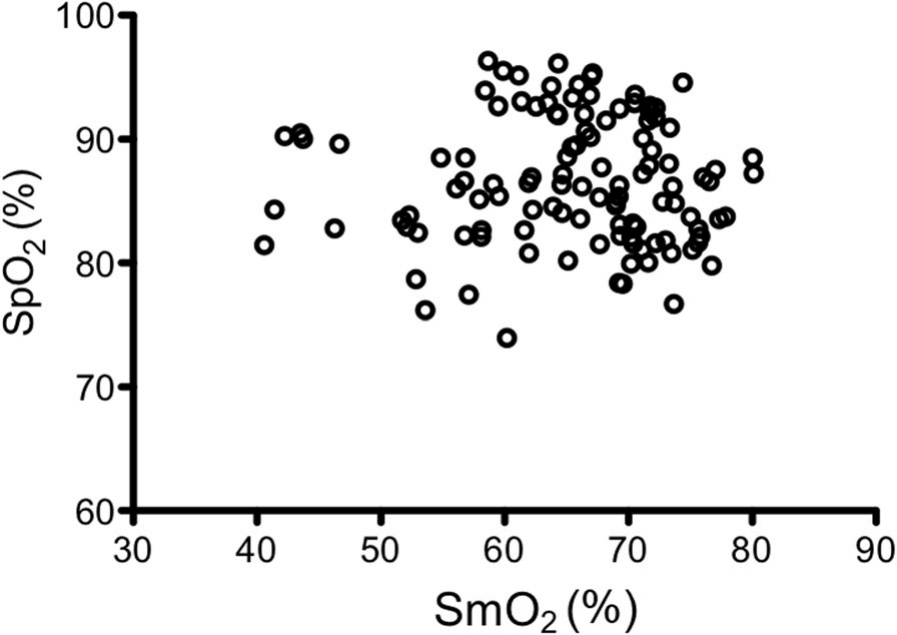
Correlation between muscle saturation of oxygen (SmO_2_) and arterial saturation of oxygen measured by pulse oximetry (SpO_2_) (r = − 0.01, not significant). Points represent SmO_2_ and SpO_2_ values collected over the 6 time-points of the hand grip exercise trial for each subject during hypoxia

Task duration and tension-time index did not differ between gases (Table 2). SmO_2_ and SpO_2_ were not correlated when the data set of one outlier (values two standard deviations greater than the mean) was excluded (r = − 0.01) (Fig. 4).

**Table 2:** Effects of normoxia and hypoxia on performance, ventilatory and cardiac parameters from intermittent handgrip exercise

	Normoxia		Hypoxia	
Task duration (s)	159.8 ± 69.8		166.6 ± 95.5	
Tension-time index (N·min)	489.3 ± 233.2		484.1 ± 263.6	
	Baseline	Task failure	Baseline	Task failure
P_ET_CO_2_ (mmHg)	34.8 ± 5.9	34.7 ± 6.6	34.1 ± 4.6	33.2 ± 4.7
SpO_2_ (%)	98.3 ± 1.4	98.7 ± 1.0	85.6 ± 4.5^*a*^	88.5 ± 6.3^*bc*^
V_E_ (L/min)	8.8 ± 3.7	11.7 ± 6.1	10.5 ± 3.3	15.0 ± 6.2^*bc*^
HR (bpm)	76.0 ± 12.3	90.8 ± 12.7^*a*^	86.7 ± 10.2^*a*^	100.0 ± 12.7^*bc*^
BP Systolic (mmHg)	121 ± 13	126 ± 10	123 ± 10	121 ± 14
BP Diastolic (mmHg)	69 ± 9	75 ± 7^*a*^	72 ± 8	75 ± 8^*c*^
Dyspnea (10-pt Borg)	0 ± 0	2 ± 2^*a*^	0 ± 0	2 ± 1^*c*^
Forearm fatigue (10-pt Borg)	0 ± 0	4 ± 2^*a*^	0 ± 0	4 ± 2^*c*^

Values are presented as mean ± SD and *n* = 20, except for measures of P_ET_CO_2_ (*n* = 17) and V_E_ (*n* = 19) in hypoxia, and V_E_ (n = 19) in normoxia. *P*_*ET*_*CO*_*2*_ partial pressure of end-tidal CO_2_, *SpO*_*2*_ arterial oxygen saturation, *V*_*E*_ minute ventilation, *HR* heart rate, *BP* blood pressure. ^*a*^ Values are significantly different from the baseline normoxic condition. ^*b*^ Values are significantly different from the task failure normoxic condition. ^*c*^ Values are significantly different from the baseline hypoxic condition

Heart rate was higher in the hypoxic condition compared to normoxia at task failure and increased compared to baseline (*p* = 0.000) (Table 2). The minute ventilation at task failure was higher under hypoxia compared to normoxia (*p* = 0.033) and higher compared to baseline (*p* = 0.001) (Table 2). No differences were detected in P_ET_CO_2_ or systolic blood pressure across trials or between gases. However, diastolic blood pressure was higher at task failure compared to baseline for both normoxic and hypoxic HGE (*p* = 0.000) (Table 2). Similarly, rating of perceived exertion for dyspnea and forearm fatigue did not differ between normoxic and hypoxic HGE but were higher at task failure compared to baseline (*p* = 0.000) (Table 2).

## Discussion

This study investigated the effects of normobaric hypoxia compared to normoxia on FDS muscle oxygenation and recruitment during a high duty:cycle intermittent HGE to task failure in healthy adults. Intermittent HGE during normobaric hypoxia compared to normoxia caused a greater decrease in FDS SmO_2_ and it remained lower throughout the duration of the hypoxic HGE to task failure. With respect to sEMG, a lower mean power frequency was demonstrated during hypoxic compared to normoxic HGE. However, despite the lower SmO_2_ and lower mean power frequency, no differences in the other NIRS variables (ΔO_2_Hb, ΔHHb, ΔTHb), task duration, tension-time index, or perceived exertion were shown between normoxic and hypoxic HGE trials. During both hypoxic and normoxic HGE, FDS O_2_Hb initially decreased from baseline to 40% of task duration, and then later increased from 40% to task failure. Conversely, HHb and tHb increased from baseline to task failure under both normoxia and hypoxia albeit the increase in tHb lagged behind changes in O_2_Hb and HHb being only significant during the last quintile of duration to task failure.

The SmO_2_ was lower throughout the hypoxic compared to the normoxic HGE and progressively decreased throughout both HGE trials. No studies to date have demonstrated decreases in SmO_2_ in the FDS during acute hypoxic HGE. However, a decrease in SmO_2_ with exercise is consistent with what has previously been shown in respiratory muscles during incremental inspiratory threshold loading and in larger muscle groups during incremental cycle ergometry and during isometric knee extension exercises [20, 32, 33]. The greater overall lowering of SmO_2_ during hypoxia in this study may be attributed to a longer wash-in period of 10 min, compared to five minutes, and a lower FiO_2_ of 12% compared to previous studies utilizing hypoxia with FiO_2_ of 15–16% [32, 33]. In this study, FDS HHb and tHb did not differ between normoxic and hypoxic trials, similar to that previously shown [20, 33]. However, as expected, both HHb and tHb increased progressively throughout the duration of exercise. A previous report utilizing HGE have demonstrated similar increases in tHb [34] and HHb [34] in the FDS. The increase in HHb is likely attributed to an increase in motor unit recruitment, as evidenced by sEMG root mean square. Additionally, increases in HHb are believed to reflect greater O_2_ extraction [35], which is expected to increase with task duration in order to meet the oxygen demands of the working muscle.

The hypoxic HGE resulted in a lower mean power frequency than normoxic HGE; however, both normoxic and hypoxic protocols increased root mean square and decreased mean power frequency in FDS at task failure compared to baseline values. The increase in root mean square and decrease in mean power frequency is consistent with previous work that investigated the neuromuscular response to fatiguing exercise [36]. Increased root mean square is indicative of greater motor unit recruitment required to produce the same desired amount of force when approaching task failure, related to increases in central drive [37, 38]. The decrease in mean power frequency indicates more synchronous firing of motor units, which has been shown to occur during fatigue [39]. Decreased firing rate of the working motor units is related to inhibitory afferents that are sensitive to changes in muscle metabolic state, which decreases central drive [36] and conduction velocity [40] when approaching task failure. Hypoxic compared to normoxic exercise has been shown to induce greater firing of type III/IV afferents in working muscle, which inhibit α motor neurons and depress central drive to diminish the production of fatigue-related metabolites [41]. However, during fatiguing exercise under moderate hypoxia (SaO_2_~82%), fatigue related metabolites have the largest influence on type III/IV muscle afferent activation, and by extension the greatest effect on central drive [41]. The combined effects of hypoxia and fatigue metabolites support the findings of a decreased mean power frequency in the hypoxic condition compared to the normoxic condition.

With regard to FDS O_2_Hb, this study found that it initially decreased to the mid-point of HGE duration followed by an increase to task failure although FDS O_2_Hb never returned to baseline under the hypoxic condition. This pattern of an increase midway through the exercise trial is contrary to previous studies that have shown progressive decreases in O_2_Hb throughout trial durations of exercise [20, 42, 43]. To date, only one study similarly demonstrated a decrease with a subsequent increase in O_2_Hb in the gastrocnemius during a heel-raising exercise regime [44]. The initial decrease in O_2_Hb could be attributed to greater oxygen utilization than delivery during the beginning stages of the HGE task [35] whereas with increased HGE duration, a rise towards baseline in O_2_Hb could be due to the progressive increase in heart rate and local exercise induced vasodilation. The progressive elevated heart rate subsequently increased perfusion and oxygen delivery, at least in part, compensated for the increased O_2_ extraction. Of no surprise, there was no correlation between SmO_2_ and SpO_2_, indicating that changes in SpO_2_ are not reflective of changes in muscle oxygenation as has been previously shown [45, 46].

No difference was found in task duration or tension-time index between hypoxic versus normoxic HGE trials. Previous literature using intermittent isometric exercise has shown an decreased endurance time under hypoxic conditions in small muscle groups compared with normoxic conditions [5, 6]. The current experimental protocol was likely unable to detect differences in task duration between the hypoxic and normoxic trials due to the relatively high workload and duty cycle utilized (50% MVC and 2 s contraction: 1 s relaxation). This intensity and duration of the HGE protocol may have predominantly challenged anaerobic glycolytic versus aerobic pathways. Performances of high intensity exercise can activate anaerobic glycolytic pathways for up to three minutes [47], a length of time similar to task duration in this study. This dependence on anaerbiosis may have been facilitated by the relatively high proportion of type II fibres in the FDS (41%) [48], which is much higher that the 20% reported in the adductor pollicis [49]. The exercise protocols in previous reports likely had greater involvement of aerobic pathways based on the fiber type proportion of the adductor pollicis [49], work to rest ratios (5 s on, 5 s off), and longer task durations [8, 9, 18]. The finding of no difference in task duration under hypoxia in this study is more consistent with studies using sustained isometric exercise. In such experimental protocols the higher intensity and sustained task resulted in muscles fatiguing faster [9, 20, 50], which was reflected in shorter task durations within anaerobic glycolytic thresholds.

The usage of NIRS and EMG together as demonstrated in this study is a promising non-invasive tool for simultaneously evaluating skeletal muscle oxygenation and muscle activation, together providing a more comprehensive evaluation of muscle performance. This has particular relevance in athletic populations and sports setting. It is well established that strength and endurance performance of forearm muscles is an important characteristic of climbers [51]. Previous studies have examined the impact of diverse exercise protocols on muscle oxygenation profiles in rock and ice climbers and furthermore have looked at differences across a range of athletic abilities [16–18, 50]. Further investigation into how muscle activation and oxygenation profiles change in response to acute hypoxic stress may aid in further characterizing and identifying the physiological differences, adaptations and limitations that occur in athletes of varying backgrounds—from healthy controls to elite athletes.

While NIRS is a reliable, non-invasive tool for the examination of muscle oxygenation, there are limitations with respect to the utilization of this device. First, NIRS is unable to differentiate between chromophores of Hb and myoglobin due to similar light absorption properties, thus values are reflective of changes in both. Additionally, NIRS optodes were placed on a single point on the FDS, and thus may not be entirely representative of other regions of the working muscle, especially in those with a larger FDS. Finally, this study examined healthy young participants, which should be taken into consideration when relating findings from this study to older or diseased populations.

## Conclusions

In conclusion, this study demonstrated that acute exposure to normobaric hypoxia results in a greater decline in SmO_2_ and a greater level of muscle fatigue as evidenced by decreased mean power frequency of the FDS during a high duty cycle intermittent HGE to task failure. Although mean power frequency was lower during hypoxia compared to the normoxic condition, this did not result in a shorter duration, lower tension-time index to task failure or greater changes in ΔO_2_Hb, ΔHHb, ΔtHb. Taken together, a lower SmO_2_ during hypoxia at rest did not accentuate the rate of decline by HGE compared to the normoxic HGE. In addition, similar ΔHHb occurred during both gas conditions of the HGE indicative of similar oxygenation utilization. Thus, hypoxia affected the resting SmO2 but did not accentuate any further changes during exercise compared to normoxic HGE. Overall, the utilization of NIRS and sEMG simultaneously can provide a better understanding of both the degree of muscle activation and changes in metabolic activity and thus provide a more holistic understanding of physiological changes occurring in the working muscle.

## Data Availability

The datasets generated and/or analysed during the current study are not publicly available due proprietary software but are available from the corresponding author on reasonable request.

## Abbreviations

FDS: Flexor digitorum superficialis
FiO_2_: Inspired fractional concentration of oxygen
HGE: Submaximal intermittent handgrip exercise
HHb: Deoxygenated hemoglobin
O_2_Hb: Oxygenated hemoglobin
sEMG: Surface electromyography
SmO_2_: Oxygen saturation of muscle
SpO_2_: Arterial oxygen saturation via pulse oximetry
tHb: Total hemoglobin
